# Demographic and clinical characteristics associated with advanced stage colorectal cancer: a registry-based cohort study in Saudi Arabia

**DOI:** 10.1186/s12885-024-12270-1

**Published:** 2024-04-26

**Authors:** Norah Alsadhan, Sultana A Alhurishi, Mar Pujades-Rodriguez, Farag Shuweihdi, Cathy Brennan, Robert M West

**Affiliations:** 1 Leeds Institute of Health Sciences, School of Medicine, University of Leeds, Leeds, UK; 2 Department of Community Health Sciences, College of Applied Medical Sciences, King Saud University, Riyadh, Kingdom of Saudi Arabia; 3 Dental Translational & Clinical Research Unit, School of Dentistry, University of Leeds, Leeds, UK; 4 Psychological & Social Medicine, School of Medicine, University of Leeds, Leeds, UK

**Keywords:** Colorectal cancer, Late-stage, Risk factors, Characteristics, Decision tree, Saudi Arabia

## Abstract

**Background:**

In Saudi Arabia, approximately one-third of colorectal cancer (CRC) patients are diagnosed at an advanced stage. Late diagnosis is often associated with a worse prognosis. Understanding the risk factors for late-stage presentation of CRC is crucial for developing targeted interventions enabling earlier detection and improved patient outcomes.

**Methods:**

We conducted a retrospective cohort study on 17,541 CRC patients from the Saudi Cancer Registry (1997–2017). We defined distant CRCs as late-stage and localized and regional CRCs as early-stage. To assess risk factors for late-stage CRC, we first used multivariable logistic regression, then developed a decision tree to segment regions by late-stage CRC risk, and finally used stratified logistic regression models to examine geographical and sex variations in risk factors.

**Results:**

Of all cases, 29% had a late-stage diagnosis, and 71% had early-stage CRC. Young (< 50 years) and unmarried women had an increased risk of late-stage CRC, overall and in some regions. Regional risk variations by sex were observed. Sex-related differences in late-stage rectosigmoid cancer risk were observed in specific regions but not in the overall population. Patients diagnosed after 2001 had increased risks of late-stage presentation.

**Conclusion:**

Our study identified risk factors for late-stage CRC that can guide targeted early detection efforts. Further research is warranted to fully understand these relationships and develop and evaluate effective prevention strategies.

**Supplementary Information:**

The online version contains supplementary material available at 10.1186/s12885-024-12270-1.

## Introduction

Colorectal cancer (CRC) is the third most common type of cancer and the second leading cause of cancer-related deaths worldwide, despite existing prevention strategies to lower its risk [1]. According to the latest incidence report from the Saudi Cancer Registry (SCR), CRC is the most commonly diagnosed cancer in men and the third in women. In 2020, 1,729 cases were diagnosed, accounting for almost 12.3% of all newly diagnosed cancers, and around 26% of diagnosed patients had distant CRC [2].

The Surveillance, Epidemiology and End Results (SEER) Summary Stage System classifies cancer stage based on the tumor’s potential impact on prognosis and survival, distinguishing between: (i) localized cancer, which is contained to the site of origin, with no evidence of adjoining invasion or metastasis beyond the organ; (ii) regional cancer, with involvement of local lymph nodes, tissues, or other organs; and (iii) distant cancer, which has spread to parts of the body distant from the organ of origin [3]. The prognosis of CRC largely depends on the stage at diagnosis [4]. Five-year survival rates for patients with localized and regional cancers are approximately 91% and 72%, respectively, while the survival rate for patients with distant-stage CRC is 13% [5]. Treatment costs are also considerably higher for distant CRC [6]. Therefore, identifying and targeting preventive interventions to patients and populations most prone to present with distant CRC is essential for improving survival and reducing healthcare costs.

Previous studies, primarily conducted in Western countries, have reported risk factors associated with the late-stage diagnosis of CRC. These factors include age, sex, race/ethnicity, marital status, geographic regions, family history of CRC, and cancer site [7–13]. However, results from published studies are inconsistent, highlighting the necessity for a deeper examination of these factors and their potential role in targeting CRC prevention strategies in different settings. Limited information is available in the Saudi context.

In 2016, Saudi Arabia initiated its 2030 vision, targeting strategic objectives across various sectors, including healthcare transformation under the Health Sector Transformation Program [14]. This program seeks to restructure the health sector by improving service quality, access, and disease prevention. A colorectal cancer control initiative was developed under this program. As a result, the Ministry of Health introduced the first Saudi National CRC screening program targeting individuals aged 45 and above [15]. It has been postulated that the effectiveness of such programs could be enhanced by identifying subpopulations at risk and subsequently adopting a strategic, targeted approach to screening and symptom-awareness campaigns [16]. Adopting CRC screening among the public is anticipated to improve early detection rates and facilitate timely interventions and treatment, potentially reducing the CRC burden for patients, their relatives, and the healthcare system. This study will advance the knowledge of CRC in Saudi Arabia by assessing risk factors for late-stage presentation. Regional, sex, and age dependent differentials in risk factors were also examined.

## Methods

### Study design and data source

This is a retrospective cohort study using anonymized data from the SCR. Established in 1994, the registry collects cancer data nationwide from governmental and private health institutions. Data includes demographics (i.e., age, sex, marital status, and region) and tumor characterization (i.e., date of diagnosis, primary site, stage, and basis of diagnosis). The registry’s main office undertakes quality control procedures, including data verification and case linkage [17]. Cancer topography (primary site) and morphology (histology) from CRC neoplasms are coded using the second and third editions of the International Classification of Diseases for Oncology (ICD-O-2 for cancers diagnosed between 1994 and 2000 and ICD-O-3 after 2000). For CRC, coding is identical in both ICD-O versions [18].

### Study population

The study used registry data from all Saudi patients diagnosed with malignant CRC between 1997 and 2017. Colon cancer was defined as a diagnosis with any of the following topography codes: cecum (C18.0), appendix (C18.1), ascending colon (C18.2), hepatic flexure of colon (C18.3), transverse colon (C18.4), splenic flexure of colon (C18.5), descending colon (C18.6), sigmoid colon (C18.7), overlapping lesion of colon (C18.8), and colon, not otherwise specified (NOS; C18.9). Cancer of the rectosigmoid junction and rectum, NOS were defined by codes C19.9 and C20.9, respectively.

### Study outcome

The study outcome was late-stage CRC diagnosis, which was defined as distant CRC. Localized and regional CRCs were categorized as early-stage CRC.

### Covariates

**Fig. 1:**
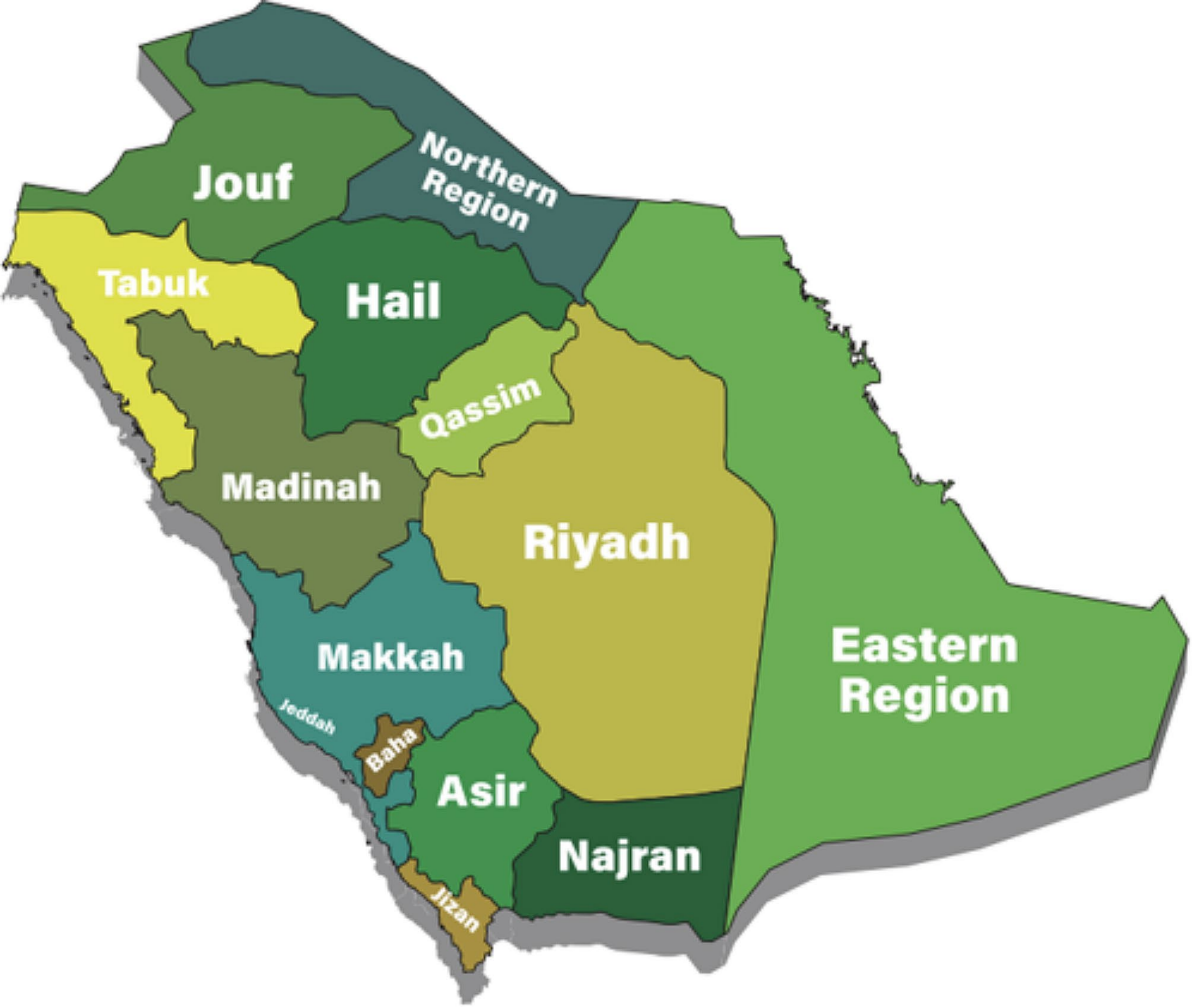
Regions of Saudi Arabia [2]

Risk factors considered included age at diagnosis (< 40, 40–49, 50–59, 60–69, 70–79, and 80 + years), sex, marital status (married and unmarried, which comprised single, divorced, and widowed individuals), region (each of the 13 administrative Saudi Arabian regions; Fig. 1), diagnosis date (grouped into four 5-year intervals), and tumor site (colon, rectosigmoid, and rectal). The registry recorded age as a continuous variable, but we categorized it into six groups for this analysis to account for nonlinear effects.

### Statistical analyses

We used summary statistics to describe the frequency and percentage of CRC patients according to disease stage at diagnosis. We then assessed the association between cancer stage at diagnosis and patients’ demographic and clinical characteristics using multivariable logistic regression. The initial model included all study covariates. We chose the 50–59 age group as the reference category in our analysis, based on the epidemiological literature defining young-onset CRC as diagnoses before age 50 and our interest in assessing ratios for patients diagnosed at younger ages [19, 20].

In a second stage, we stratified the model by sex to examine potential sex differences in risk factors. The unequal sample sizes of the 13 regions posed a methodological challenge for stratifying our logistic regression analyses by region. Thus, we used Fast and Frugal Trees (FFT) [21], a type of decision tree analysis, to identify regions that could be grouped according to their risk of developing late-stage CRC, allowing for more statistically robust analyses. Detailed explanation of the FFT method is provided in the Supplemental Methods File. We finally assessed associations with late-stage diagnosis within each identified geographical group of similar CRC risk profile, stratified by sex, to quantify variation in risk factors.

We used multiple imputation by chain equations, generating ten datasets, to impute covariate data. There were 1,922 (9.9%) CRC patients with missing stage information. In primary analysis, we performed a complete stage-data analysis to prevent potential bias introduction associated with outcome imputation. To assess the robustness of the estimates, we also imputed missing stage data using multiple imputation with chained equations as a sensitivity analysis. Detailed explanation of the imputation method and the handling of missing covariate data in multivariable logistic regression and FFT analyses is provided in the Supplemental Methods File.

All analyses were conducted in *R 4.3.2* [22], including the “FFTrees” package for the decision tree classifier [21]. *P*-values were two-sided. Results with *P*-values less than 0.05 were considered statistically significant. We used the likelihood ratio test to assess the overall significance of each risk factor considered.

## Results

A total of 19,463 new cases of CRC were registered during the study period, and 17,541 of them had known CRC stage. Of cases with recorded CRC stage, 98.8% were morphologically verified (MV), and only 50 (0.3%) were identified through death certificate only (DCO). In contrast, among the 1,922 cases with unknown CRC stage, 83.6% were MV, and 12.5% had been identified through DCO. There were 5,139 (29%) patients with late-stage presentation (Table 1). We observed consistent demographic characteristics across both early and late-stage CRC diagnoses. The overall mean age was 58 years. Most patients were males, married, lived in Riyadh, and had colon cancer.

**Table 1:** Distribution of patient characteristics at diagnosis by disease stage and associated adjusted odds ratios for late versus early-stage CRC presentation

Characteristics		Early-stage N (%)	Late-stage N (%)		OR (95%CI)
		12,402 (70.7)		5,139 (29.3)	
Age in yrs, mean (SD)		57.93 (14.8)		57.81 (14.9)	
Age group in yrs	0–39	1383 (11.2)		598 (11.6)	1.10 (0.99, 1.22)
	40–49	2103 (17.0)		936 (18.2)	1.13 (1.03, 1.23)
	50–59	3113 (25.1)		1222 (23.8)	1.00 *P =* 0.01
	60–69	2941 (23.7)		1170 (22.8)	1.03 (0.93, 1.12)
	70–79	2013 (16.2)		859 (16.7)	1.11 (1.00, 1.21)
	80+	843 ( 6.8)		353 ( 6.9)	1.09 (0.95, 1.23)
Sex	Male	6930 (55.9)		2713 (52.8)	1.00 *P <* 0.001
	Female	5472 (44.1)		2426 (47.2)	1.12 (1.06, 1.19)
Marital status	Married	10,020 (89.9)		4169 (88.0)	1.00 *P <* 0.01
	Unmarried	1126 (10.1)		568 (12.0)	1.09 (0.98, 1.19)
Region	Riyadh	3787 (30.8)		1583 (31.1)	1.00 *P <* 0.0001
	Eastern	2031 (16.5)		961 (18.9)	1.13 (1.03, 1.23)
	Makkah	3150 (25.6)		1200 (23.6)	0.91 (0.83, 1.00)
	Madina	705 ( 5.7)		235 ( 4.6)	0.81 (0.65, 0.96)
	Asir	911 ( 7.4)		346 ( 6.8)	0.91 (0.77, 1.05)
	Jazan	239 ( 1.9)		77 ( 1.5)	0.77 (0.51, 1.04)
	Najran	115 ( 0.9)		53 ( 1.0)	1.09 (0.76, 1.42)
	Hail	224 ( 1.8)		108 ( 2.1)	1.15 (0.91, 1.39)
	Qassim	564 ( 4.6)		250 ( 4.9)	1.06 (0.90, 1.22)
	Baha	198 ( 1.6)		57 ( 1.1)	0.68 (0.38, 0.98)
	Jouf	86 ( 0.7)		59 ( 1.2)	1.63 (1.30, 1.97)
	Northern	67 ( 0.5)		45 ( 0.9)	1.59 (1.21, 1.98)
	Tabuk	237 ( 1.9)		118 ( 2.3)	1.18 (0.95, 1.41)
Diagnosis date	1997–2001	1112 ( 9.0)		348 ( 6.8)	1.00 *P <* 0.0001
	2002–2006	2054 (16.6)		823 (16.0)	1.28 (1.14, 1.43)
	2007–2011	3336 (26.9)		1492 (29.0)	1.43 (1.30, 1.57)
	2012–2017	5900 (47.6)		2476 (48.2)	1.34 (1.21, 1.47)
Anatomical site	Colon	7513 (60.6)		3027 (58.9)	1.00 *P <* 0.01
	Rectosigmoid	1847 (14.9)		873 (17.0)	1.17 (1.08, 1.27)
	Rectal	3042 (24.5)		1239 (24.1)	1.01 (0.93, 1.09)

Note: CI: Confidence interval; N: Number; OR: Odds ratios adjusted for all factors in the table using complete stage data and multiple imputation of covariates; SD: Standard deviation. The *p*-values are derived from the overall likelihood ratio tests for association. Number and percentage of missing values relative to the dataset with known cancer stage: Age at diagnosis (*n* = 7, 0.04%); Marital status (*n* = 1658, 9.5%); Geographical region (*n* = 135, 0.8%). Percentages are presented by column to characterise patients’ profiles for patients with late-stage and early-stage CRC

### Factors associated with late-stage CRC

Age and sex were associated with late-stage CRC, with slightly higher adjusted ORs observed for the 40–49 and 70–70 age groups (1.13, 95%CI = 1.03–1.23; and 1.11, 95%CI = 1.00-1.21, respectively, overall *P* = 0.01) compared with the 50–59 group; and in women (1.12; 95%CI = 1.06–1.19; overall *P* < 0.001) compared to men (Table 1). Regional variations were also noted; with the highest estimates of late-stage diagnosis found in the Jouf, Northern, and Eastern regions (1.63; 95%CI = 1.30–1.97; 1.59; 95%CI = 1.21–1.98 and 1.13; 95%CI = 1.03–1.23; respectively, overall *P* < 0.0001) compared to Riyadh. Patients with rectosigmoid cancer had a higher risk of late-stage diagnosis (1.17; 95%CI = 1.08–1.27) than those with colon cancer, as were patients diagnosed within calendar periods following 1997–2001. Results from the sensitivity analysis using imputed cancer stage data (Supplemental Results File, Tables 1 and 2) were similar to the primary analysis findings.

**Table 2:** Adjusted odds ratios for late versus early-stage CRC presentation, by sex

Characteristics	Males (*N* = 9,643)		Females (*N* = 7,898)
	OR (95%CI)		OR (95%CI)
Age group in yrs			
0–39	0.95 (0.78, 1.12)		1.26 (1.09, 1.42)
40–49	1.04 (0.90, 1.19)		1.22 (1.08, 1.37)
50–59	1.00 *P =* 0.01		1.00 *P <* 0.01
60–69	0.95 (0.82, 1.08)		1.10 (0.96, 1.24)
70–79	1.11 (0.98, 1.25)		1.06 (0.90, 1.22)
80+	1.19 (1.00, 1.37)		0.91 (0.68, 1.14)
Marital status			
Married	1.00 *P =* 0.32		1.00 *P =* 0.01
Unmarried	1.14 (0.94, 1.35)		1.23 (1.10, 1.37)
Region			
Riyadh	1.00 *P <* 0.0001		1.00 *P <* 0.0001
Eastern	1.21 (1.08, 1.34)		1.04 (0.90, 1.18)
Makkah	0.97 (0.84, 1.09)		0.86 (0.73, 0.99)
Madina	0.88 (0.67, 1.10)		0.71 (0.47, 0.96)
Asir	0.88 (0.69, 1.07)		0.95 (0.75, 1.15)
Jazan	0.95 (0.60, 1.30)		0.60 (0.20, 1.01)
Najran	1.35 (0.89, 1.81)		0.86 (0.37, 1.34)
Hail	1.43 (1.11, 1.74)		0.88 (0.51, 1.24)
Qassim	1.18 (0.96, 1.40)		0.93 (0.70, 1.17)
Baha	0.78 (0.34, 1.22)		0.60 (0.18, 1.02)
Jouf	2.08 (1.63, 2.52)		1.21 (0.68, 1.73)
Northern	1.16 (0.58, 1.73)		2.14 (1.60, 2.68)
Tabuk	1.25 (0.95, 1.55)		1.11 (0.76, 1.47)
Diagnosis date			
1997–2001	1.00 *P <* 0.01		1.00 *P <* 0.001
2002–2006	1.24 (1.04, 1.44)		1.33 (1.12, 1.55)
2007–2011	1.39 (1.20, 1.57)		1.49 (1.29, 1.69)
2012–2017	1.24 (1.06, 1.42)		1.48 (1.29, 1.67)
Anatomical site			
Colon	1.00 *P =* 0.03		1.00 *P =* 0.05
Rectosigmoid	1.18 (1.05, 1.30)		1.18 (1.04, 1.31)
Rectal	0.99 (0.88, 1.10)		1.04 (0.92, 1.16)

Note: CI: Confidence interval; N: Number; OR: Odds ratios adjusted for all factors in the table using complete stage data and multiple imputation of covariates. The *p*-values are derived from the overall likelihood ratio tests for association

We found an increased risk of late-stage CRC presentation in younger age (< 50) and unmarried women and in men aged 80 years or more (Table 2). We also found regional sex-related disparities in late-stage disease risk. The highest estimates were found for Jouf in men and for the Northern area in women (2.08; 95%CI = 1.63–2.52; and 2.14; 95%CI = 1.60–2.68; respectively, overall *P* < 0.0001) compared with Riyadh. Furthermore, in the Hail region, men had increased risk of late-stage presentation (1.43; 95%CI = 1.11–1.74), while no evidence of increased risk was observed amongst women (0.88; 95%CI = 0.51–1.24). Risk estimates for cancer location were similar irrespective of sex, and similar risk patterns were also found for calendar periods.

### Regional disparities in factors associated with late CRC

**Fig. 2:**
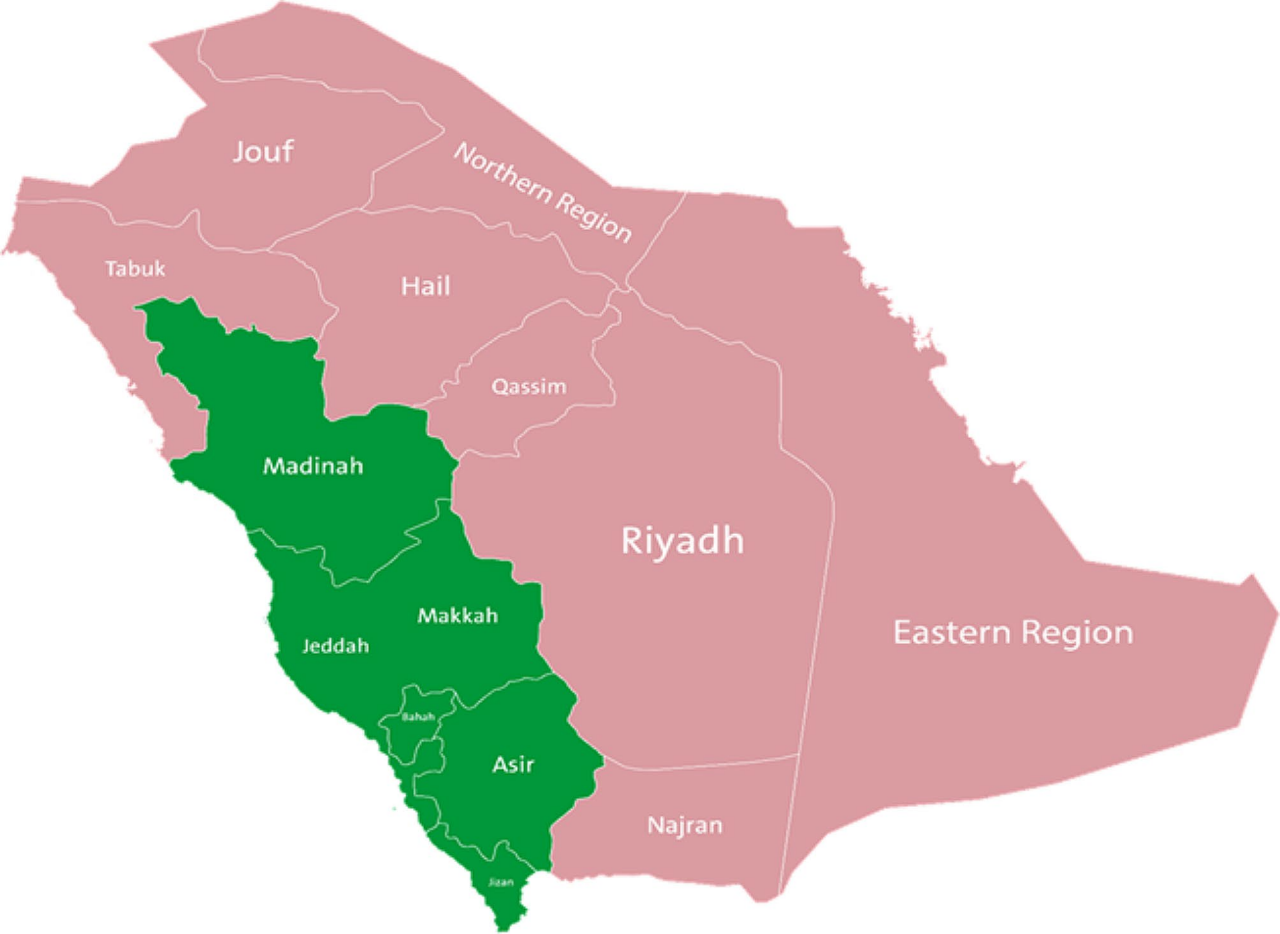
Geographic distribution of late-stage CRC risk in Saudi Arabia, based on FFT analysis: high-risk regions (Group A) are colored in red, and low-risk regions (Group B) in green

Through FFT analysis (Supplemental Results File), we defined two geographical areas based on late presentation risk (Fig. 2). Group A (high risk for late-stage CRC) included Riyadh, Eastern, Najran, Hail, Qassim, Jouf, Northern, and Tabuk regions; and Group B included Makkah, Madina, Asir, Jazan, and Baha.

In Group A, identified as high-risk, women under 50 and unmarried had an increased risk of late-stage CRC (Table 3). These differences were not found in Group B. Increased risk of late-stage rectosigmoid cancer was observed for women in Group A (1.34; 95%CI = 1.16–1.51; overall *P = <* 0.01) and for men in Group B (1.29; 95%CI = 1.09–1.49; overall *P =* 0.04). Compared to the 1997–2001 period, the risk of presenting with late-stage CRC in men and women was increased in subsequent periods across all regions. This increase was particularly pronounced for females in Region B.

**Table 3:** Adjusted odds ratios for late versus early-stage CRC presentation, by region and sex

Characteristics	Group (A) regions (*N* = 10,370)		Group (B) regions (*N* = 7,171)	
	Males (*N* = 5,658)	Females (*N* = 4,712)	Males (*N* = 3,985)	Females (*N* = 3,186)
	OR (95%CI)	OR (95%CI)	OR (95%CI)	OR (95%CI)
**Age group in yrs**				
0–39	0.95 (0.73, 1.17)	1.34 (1.14, 1.55)	0.95 (0.67, 1.23)	1.12 (0.84, 1.39)
40–49	1.02 (0.83, 1.20)	1.32 (1.14, 1.50)	1.11 (0.88, 1.34)	1.05 (0.81, 1.29)
50–59	1.00 *P =* 0.22	1.00 *P <* 0.01	1.00 *P =* 0.01	1.00 *P =* 0.02
60–69	1.01 (0.84, 1.18)	1.09 (0.91, 1.27)	0.87 (0.66, 1.08)	1.10 (0.88, 1.33)
70–79	1.11 (0.93, 1.28)	1.11 (0.90, 1.33)	1.10 (0.88, 1.32)	0.94 (0.69, 1.20)
80+	1.13 (0.88, 1.37)	1.09 (0.80, 1.38)	1.23 (0.95, 1.52)	0.63 (0.26, 1.01)
**Marital status**				
Married	1.00 *P =* 0.85	1.00 *P =* 0.02	1.00 *P =* 0.30	1.00 *P =* 0.38
Unmarried	1.07 (0.82, 1.31)	1.25 (1.09, 1.42)	1.23 (0.97, 1.49)	1.19 (0.99, 1.40)
**Diagnosis date**				
1997–2001	1.00 *P <* 0.0001	1.00 *P =* 0.02	1.00 *P =* 0.06	1.00 *P <* 0.0001
2002–2006	1.16 (0.90, 1.42)	1.18 (0.91, 1.45)	1.40 (1.08, 1.71)	1.81 (1.44, 2.18)
2007–2011	1.56 (1.32, 1.80)	1.39 (1.14, 1.64)	1.20 (0.90, 1.50)	1.85 (1.50, 2.19)
2012–2017	1.34 (1.11, 1.57)	1.28 (1.05, 1.52)	1.13 (0.84, 1.42)	2.07 (1.73, 2.41)
**Anatomical site**				
Colon	1.00 *P =* 0.47	1.00 *P <* 0.01	1.00 *P =* 0.04	1.00 *P =* 0.92
Rectosigmoid	1.09 (0.93, 1.25)	1.34 (1.16, 1.51)	1.29 (1.09, 1.49)	0.98 (0.76, 1.20)
Rectal	0.99 (0.85, 1.12)	1.04 (0.89, 1.19)	0.98 (0.86, 1.15)	1.06 (0.86, 1.26)

Note: CI: Confidence interval; N: Number; OR: Odds ratios adjusted for all factors in the table using complete stage data and multiple imputation of covariates. The *p*-values are derived from the overall likelihood ratio tests for association

## Discussion

The escalating incidence and mortality rates associated with CRC have rendered it a critical public health concern in Saudi Arabia [23, 24]. Considering the essential role of the stage at diagnosis in CRC prognosis [25], it is vital to identify patients at increased risk for late-stage CRC to enhance early detection strategies. This study is the first in Saudi Arabia to explore factors associated with late-stage CRC presentation using a large national registry database.

In our study, 29.3% of patients were diagnosed with late-stage CRC, higher than the 23% reported by the U.S. SEER program [26]. While the U.S. has benefitted from a long history of CRC screening leading to early detections [27], Saudi Arabia only recently initiated its screening program [15]. Differences in referral pathways and diagnostic timelines [28], and societal and cultural factors, including low public awareness regarding CRC screening [29, 30], might also contribute to delays in diagnosis and reduced screening uptake in Saudi Arabia.

We report a higher risk of late-stage CRC in women than men. This is consistent with previous studies in Saudi Arabia and the U.S [31, 32] but contrasts with findings from other countries. A review of U.K. national data showed no differences in the proportion of men and women diagnosed at advanced CRC stages [33]. Conversely, Nguyen et al.’s systematic review and meta-analysis, including seventeen studies conducted between 1993 and 2008 in North America, Europe, and Asia, reported a pooled estimate of 83% higher risk of advanced CRC diagnosis in men than in women [8]. The discrepancy between men and women in CRC stage presentation could arise from differences in tumor locations: men often have distal colon cancer, which is easier to detect early, while women have tumors in the harder-to-detect proximal colon [34, 35]. Our study, however, highlighted that men and women were more likely to present with distal disease at the rectosigmoid junction.

A possible explanation of the observed sex-related disparity in late-stage CRC risk could be differences in screening utilization and in psychosocial factors, such as perceived or real screening barriers. A systematic review of 134 international studies on CRC screening participation found women less likely to be screened [36], possibly due to receiving fewer physician referrals, viewing CRC as a ‘male disease’, and perceiving more barriers to screening uptake [36, 37]. A recent Saudi study supported this, as women faced more screening barriers than men, including fear and embarrassment of the screening procedure [38]. Additionally, the lower CRC incidence rates in Saudi women compared to men may also perpetuate the view of CRC as predominantly a male disease [2, 23]. This perception could thereby affect screening uptake and early diagnosis among Saudi women.

Another plausible explanation may be the existence of gender disparities in healthcare access and provision. Gender bias in clinical care, particularly in cardiovascular disease and chronic pain treatment, has been well documented [39–41]. Prior research indicated that women, more often than men, are less likely to receive adequate pain management. Evidence suggests that women’s complaints are often dismissed as emotional or of psychogenic origin, potentially leading to delayed diagnoses compared to men [39]. Although this issue, to our knowledge, has not been explored within the context of colorectal cancer, its potential impact cannot be overlooked, and further research into this area is needed. Alcalde-Rubio’s recent review underlines that health systems and providers continue to neglect gender disparities in healthcare and that developing gender-oriented intervention strategies and training of healthcare providers is essential to address and mitigate these biases effectively [42].

Our results revealed that young women are at an increased risk of late-stage CRC diagnosis, consistent with prior research linking CRC in people under the age of 50 to aggressive tumor characteristics [43–46]. Yet, a direct link between young women and advanced CRC is not established. CRC in the young might involve diagnostic delays due to misattributing symptoms to benign conditions [47]. We also hypothesize that factors like health-seeking behaviors, cultural perceptions, patient-practitioner communication, and CRC screening practices, which have also been emphasized in previous studies [31, 48], might affect late-stage diagnoses in young women, highlighting the need for more detailed research to explore these potential associations. While the current Saudi guidelines for initiating CRC screening at the age of 45 align with current recommendations in the U.S [49, 50], it’s important to note that these guidelines were formulated based on limited Saudi data. Our findings emphasize the potential advantage of starting screenings at 45. Yet, they also underscore the need for further research into the increased risk among women under 45 years and potential consideration of gender-specific recommendations for the age of initiation of CRC screening to achieve earlier and more effective detection and treatment of CRC amongst women.

Unmarried women, but not men, had a higher risk of late-stage CRC. This finding is consistent with a recent systematic review of 18 studies, mainly from the U.S., indicating the positive effect of marriage on the likelihood of presenting with early-stage cancer [13]. In their analysis of about 1,26 M patients with major cancers, including CRC, Aizer et al. found that unmarried patients, across all cancers, were more likely to be diagnosed with advanced stage cancer compared to those who were married [51]. A previous study also highlighted that being unmarried is associated with delayed CRC diagnosis, resulting in more advanced stages at presentation [52]. This observation may arise from a higher financial status, facilitating access to healthcare services [13, 53].

Additionally, married individuals often benefit from emotional and informational support from their spouses, promoting positive health-related behavior, including regular medical check-ups and greater use of screening services [51, 54, 55]. Cross-sectional studies in Saudi Arabia have also reported higher knowledge of CRC and its screening among married individuals [56, 57]. While CRC screening is freely available in Saudi Arabia, access alone does not ensure equitable utilization. Further research is needed to confirm and further explain observed disparities in cancer outcomes based on marital status. Exploring unique barriers and concerns in accessing healthcare for unmarried women is also essential for developing targeted awareness and social support interventions and training for healthcare providers to recognize and address any potential related biases in the diagnosis process.

Emerging evidence suggests that the anatomical location of CRC impacts the prognostic characteristics of the disease [58]. Some studies noted differences in tumor biology, clinical presentation, and outcomes between proximal (right) and distal (left) CRC [12, 59, 60]. These differences were usually attributed to the distinct embryologic origins, gross macroscopic pathology, and metastatic patterns of the right and left colon segments [12, 61, 62]. In our study, rectosigmoid cancers in men and women were more likely to be diagnosed at a late stage compared to colon cancers. This aligns with findings from a recent registry-based study of 25,282 Australian CRC patients that showed an association between distal tumors and presentation at a distant CRC stage [46]. Saudi Arabia lacked established guidelines for CRC screening modalities and frequency during our study period. However, the recently introduced Saudi National CRC screening program recommends the Fecal Immunochemical Test (FIT) for asymptomatic individuals at average risk and colonoscopy for those at higher risk [15]. The FIT test has greater sensitivity in detecting left-sided colon lesions [63, 64]. Therefore, given the increased risk of late-stage CRC associated with rectosigmoid cancers identified in our study, these findings reinforce the recommendation for using immunochemical testing methods for early detection.

Our findings showed an increased risk of late-stage CRC presentation in recent years, contrasting with findings from countries with well-established screening programs. For example, Vather et al. suggested that Australia’s enhanced CRC screening uptake could be linked to a recent decline in advanced CRC diagnoses [46]. There were no national screening guidelines or awareness initiatives in Saudi Arabia during the study period; therefore, it is not possible to correlate screening implementation with CRC trends. Plausible explanations for our findings might be an increasing proportion of patients diagnosed before death (who might have been missed in earlier years) or advancements in the documentation and reporting of CRC stage over time. It is essential to analyze trends in late-stage diagnosis following the launch of the Saudi CRC screening program to ascertain its effectiveness and the impact of screening on disease presentation.

Previous studies in Saudi Arabia have primarily focused on the incidence of CRC, with findings indicating the highest disease rates in the Riyadh and Eastern regions, possibly due to the high population density in these areas [65]. However, no previous studies have investigated the disparities in colorectal cancer outcomes and stages across different regions. The unconventional approach of utilizing FFT analysis for regional grouping was chosen to address the variability in regional sample sizes. Yet, decision trees are considered a powerful decision-making tool for identifying distinctive homogeneous subgroups to develop tailored interventions [66, 67]. It also recognizes interactions between factors- a distinctive feature often overlooked in other methods to simplify analysis [68]. In our FFT analysis, region was identified as the primary influencing factor on disease stage, while sex was recognized as the second most significant factor (see Supplemental Results File). This highlights the critical need to account for geographical and sex-specific factors when examining CRC stage at presentation.

In Saudi Arabia, free oncology care is provided to all nationals through public cancer facilities concentrated mainly in Riyadh, Makkah, and the Eastern regions [69]. Though we anticipated these centralized resources would result in earlier CRC stage presentations in these areas compared to other regions, our results showed otherwise. Patients in Makkah, Madina, Asir, Jazan, and Baha were at a lower risk of presenting with late-stage CRC, whereas those in Riyadh, Eastern, Najran, Hail, Qassim, Jouf, Northern, and Tabuk regions faced an increased risk of late-stage CRC. Because of the centralization of cancer facilities, there might be a travel barrier for patients referred from other areas for further diagnostic confirmation, potentially resulting in diagnostic delays. Alahmadi et al. emphasized this viewpoint by noting that the extended travel times to cancer facilities in Saudi Arabia’s Northern and Southern regions might contribute to worse cancer outcomes [69]. This hypothesis, however, contrasts with our findings, mainly as Jazan and Baha patients (categorized as Southern regions by Alahmadi et al. [69]) had a lower likelihood of a late-stage disease than Riyadh patients.

There are several challenges in explaining the regional differences in disease stage observed in our study. The Saudi National CRC screening initiative was established after 2017, and our data reflected the period from 1997 to 2017. CRC screening was opportunistic during this period, mainly based on healthcare providers’ recommendations and referrals [70]. A study conducted in 2014, including 130 family physicians in Riyadh, found that 56% did not recommend CRC screening despite a positive attitude [71]. Given that this study was confined to Riyadh and lacked comparative data from other regions, it remains unclear whether this trend is specific to Riyadh and contributes to its increased rates of late-stage CRC or if it mirrors a more widespread pattern across the other regions. Additionally, several Saudi studies showed a general lack of awareness about CRC and its screening that was not confined to any particular region [30, 56, 72, 73].

There is limited Saudi evidence on screening rates and modalities by geographical area during the study period. The only Saudi national study that assessed CRC screening use across all 13 administrative regions surveyed 2,945 individuals over 60 years old from 2006 to 2007. This study found a low screening prevalence of 5.6% but did not provide region-specific data. Within this cohort, the fecal occult blood test was administered to 4.4% of subjects, while endoscopic procedures were performed in only 0.6% [74].

Behavioral risk factors like low physical activity and smoking, and obesity are known risk factors for CRC [75]. Their association with a worse disease prognosis has been suggested [76, 77], as well as with CRC incidence in the Saudi population [78, 79]. However, whether variations in the prevalence of these factors across regions could explain the observed discrepancies in late-stage CRC presentation cannot be established. The 2019 Saudi World Health Survey included 10,000 households across all 13 administrative regions and assessed behavioral risk indicators, healthcare system satisfaction, and chronic disease prevalence. While regional differences in behavioral risk factors and health indicators were reported, these did not correlate with our study findings on late-stage disease presentation. For example, while the Baha region showed a lower risk of late-stage CRC in our study, the survey indicated high smoking and obesity rates and poor dietary habits in that region [80].

Multiple factors might contribute to explaining the observed regional differences in CRC stage at diagnosis. One key factor is the disparity in quality, access, and utilization of primary healthcare services across regions, which is vital for early detection. A comprehensive review of studies assessing primary healthcare services in Saudi Arabia highlighted issues such as limited access and poor effectiveness in managing chronic diseases, patient-doctor interactions, and health education. Communication barriers with non-Arabic speaking care professionals further increased these difficulties. Regional disparities were not reported in this study [81]. In 2017, Alfaqeeh et al. examined primary healthcare access and utilization disparities between urban and rural areas in Riyadh province, revealing significant healthcare inequalities. Rural populations faced more barriers, including distance to health centers and limited availability of health promotion and prevention services [82]. These disparities may partly account for our study’s observed higher incidence of late-stage CRC in Riyadh.

Additionally, cultural factors such as stigma and health-seeking behaviors could vary regionally, impacting diagnosis stages [83–85]. Issues related to inconsistencies in data collection and reporting methods across regions might also play a role. A more in-depth investigation into these aspects is needed to fully understand the causes of these regional variations and develop effective intervention strategies.

Our decision tree analysis also identified a higher risk of late-stage CRC among younger, unmarried women in specific regions. These findings underscore the need to understand unique regional factors, including health practices, barriers to healthcare access, and experiences related to oncology service referrals among this demographic. Such understanding is vital for developing targeted awareness initiatives and enhancing screening uptake in these areas. Similarly important is improving access to screening facilities through primary healthcare services and training primary care providers to effectively identify and refer at-risk individuals, thereby optimizing early detection strategies.

### Limitations

While this study provides valuable insights into factors associated with CRC presentation, it is important to acknowledge certain study limitations. The SCR database lacks data on genetic syndromes, family history, lifestyle habits, and comorbidities, all essential factors in understanding CRC dynamics. Additionally, to help interpret the findings in our study, there is a lack of comprehensive Saudi data regarding screening rates, diagnostic delays, and health-seeking behaviors, both on a national scale and across different regions. Data collection on potential CRC risk factors at SCR registration could help better understand predictors of late-stage CRC at diagnosis and monitor changes over time.

The higher likelihood of late-stage presentation at the rectosigmoid junction reported in our study should be interpreted with caution, given that this site is the least common with a notably small sample size, which may limit the generalizability of this finding. The concentration of specialized cancer care facilities in Riyadh, Eastern, and Makkah, possibly led to overrepresentation of cases in these regions, potentially influencing findings from the regional comparisons.

Finally, using registry data involves inherent limitations related to potential coding errors, missing information, or inconsistencies in data collection and reporting methods across regions, particularly in early periods. Excluding 1,922 CRC cases due to missing stage information from the primary analysis, particularly from 1997 to 2001, could potentially lead to an underestimation of late-stage diagnoses during this early period. However, our sensitivity analysis showed consistent findings when missing stage data were imputed.

## Conclusion

Our study identified risk factors for late-stage CRC that can guide targeted early detection efforts, particularly for younger women in specific regions. A deeper exploration of attitudes and barriers to CRC screening, especially among women, is crucial to enhancing screening uptake and awareness in this high-risk group. As the risk of late-stage CRC presentation has increased in recent years, future research should evaluate the effectiveness of the CRC screening program and its impact on disease stage at diagnosis.

### Electronic supplementary material

Below is the link to the electronic supplementary material.

Supplementary Material 1: Supplemental Methods File

Supplementary Material 2: Supplemental Results File

## Data Availability

The datasets used and/or analysed during the current study are available from the corresponding author on reasonable request.

## Abbreviations

CRC: Colorectal cancer
CI: Confidence interval
DCO: Death certificate only
FFT: Fast and Frugal Trees
FIT: Fecal immunochemical test
ICD-O: International Classification of Diseases for Oncology
MV: Morphologically verified
N: Number
OR: Odds ratio
SCR: Saudi Cancer Registry
SD: Standard deviation
SEER: The Surveillance, Epidemiology, and End Results
