# Supplementary material for: Demographic and clinical characteristics associated with advanced stage colorectal cancer: a registry-based cohort study in Saudi Arabia
Source: BMC Cancer. 2024 Apr 26;24:533. doi: 10.1186/s12885-024-12270-1 (PMC11055310; doi:10.1186/s12885-024-12270-1)
Supplement: Supplementary file 1 — Supplementary Material 1: Supplemental Methods File [file 12885_2024_12270_MOESM1_ESM.docx]

**Additional file 1**

**Supplemental Methods (p.1,2)**

**Fast and Frugal Trees (FFT):**

Fast and Frugal Trees (FFT) is a specialized decision tree model designed to make quick, efficient decisions with limited resources. FFT analysis follows a heuristic approach, optimizing cues based on metrics such as classification accuracy, sensitivity, or specificity. The tree-building process starts by selecting the cues (predictor) with the highest accuracy. This predictor is then used to create the first decision node, and the process is repeated recursively until no further improvement in classification accuracy is possible. The result is a simple, interpretable tree that makes classifications based on a small number of decision rules (1). To implement FFT analysis, we used the “FFTrees” package in R, maintaining the package's default settings, specifically, goal.chase = NULL, max.levels = NULL, and sens.w = 0.5.

**Handling of missing covariate data:**

Three covariates had missing data. Marital status had the highest percentage of missing values (n=1658, 9.5%), followed by region (n=135, 0.8%) and age at diagnosis (n=7, 0.04%). While we were unable to empirically verify if the missing values of covariates were missing at random (MAR), we operated under the assumption that they were MAR. Consequently, we handled the missingness using the multiple imputation by chained equations (MICE) technique. Implemented in R, we generated ten imputed datasets. Each of these datasets underwent up to 50 iterations. The imputation method employed was Predictive Mean Matching, a non-parametric technique known for generating plausible imputations, as the replaced values are actual observed responses in the dataset. To ensure the reproducibility of our results, a random seed of 500 was set (2). Rubin's Rules (3) were used to combine statistical estimates (e.g., coefficients, standard errors) from analyses performed on each separate imputed dataset to yield a single combined estimate that incorporates the uncertainty introduced by the imputation process. For the FFT analysis, we conducted separate analyses on ten imputed datasets. Upon comparing the decision splits across these datasets, they demonstrated consistent results. Therefore, we reported the model results based on the first imputed dataset.

**Imputation of missing outcome data in sensitivity analysis:**

There were 1,922 (9.9%) CRC patients with missing stage information. In primary analysis, we performed a complete stage-data analysis to prevent potential bias introduction associated with outcome imputation.

Examination of the pattern of missingness throughout the study period revealed fluctuations over time, with the highest percentage of missing stage data observed in the earliest calendar period (Supplemental Results File, Figure 1). When comparing characteristics of patients with and without missing stage data (Supplemental Results File, Table 1), the eldest patients (80+) had markedly lower stage data completeness. We, therefore, conducted a sensitivity analysis by simultaneously imputing missing stage data and covariates using multiple imputations with chained equations, generating ten datasets.

**References**

1. Phillips ND, Neth H, Woike JK, Gaissmaier W. FFTrees: A toolbox to create, visualize, and evaluate fast-and-frugal decision trees. Judgment and Decision Making. 2017;12(4):344-68.

2. Van Buuren S. Flexible imputation of missing data: CRC press; 2018.

3. Rubin DB. Multiple imputation for nonresponse in surveys: John Wiley & Sons; 2004.
