# Supplementary material for: Demographic and clinical characteristics associated with advanced stage colorectal cancer: a registry-based cohort study in Saudi Arabia
Source: BMC Cancer. 2024 Apr 26;24:533. doi: 10.1186/s12885-024-12270-1 (PMC11055310; doi:10.1186/s12885-024-12270-1)
Supplement: Supplementary file 2 — Supplementary Material 2: Supplemental Results File [file 12885_2024_12270_MOESM2_ESM.docx]

**Additional file 2**

**Supplemental Results (p.1-7)**

**Table of Contents**

[**Decision Tree Analysis** 2](#_Toc163916593)

[**Tables** 3](#_Toc163916594)

[**Table 1.** Characteristics of patients with known and unknown disease stage at CRC diagnosis 3](#_Toc163916595)

[**Sensitivity analysis using multiple imputation for stage data in logistic regression** 4](#_Toc163916596)

[**Table *2*.** Adjusted odds ratios for late versus early-stage CRC presentation (N=19,463) 4](#_Toc163916597)

[**Table *3*.** Adjusted odds ratios for late versus early-stage CRC presentation, by sex (N=19,463) 5](#_Toc163916598)

[**Figures** 7](#_Toc163916599)

[**Figure 1.** Percentage of missing stage data for CRC in the Saudi Cancer Registry between 1997 and 2017 7](#_Toc163916600)

[**Figure 2.** A fast-and-frugal tree (FFT) for classifying patients as having late or early-stage CRC 7](#_Toc163916601)

# **Decision Tree Analysis**

**Results**

In the Fast and Frugal Tree (FFT) analysis, presented in Figure 2, each node is represented as a rectangle and denotes a predictor variable. Two branches extend from each node, where either one or both branches is an exit branch leading to a leaf (circle). Leaves are where decisions are made. Our FFT analysis indicated that if patients reside in the Riyadh, Eastern, Najran, Hail, Qassim, Jouf, Northern, and Tabuk regions, they were classified as having a late-stage disease. If not, they will be classified based on sex, with women having a late-stage CRC and men classified based on age. If men were younger than 50 or older than 70, they were more likely to have late-stage disease. If not, they will be further classified based on marital status, with unmarried men usually presenting with distant disease.

The FFT analysis provided further insights into the structure of our dataset. The primary node that emerged was the geographical region, highlighting its substantial influence on explaining the observed heterogeneity of our data. Regions were categorized into two separate groups based on this geographical division. Group A (high risk for late-stage CRC) included Riyadh, Eastern, Najran, Hail, Qassim, Jouf, Northern, and Tabuk regions, while Group B encompassed Makkah, Madina, Asir, Jazan, and Baha. Sex was the second node identified in the FFT analysis, highlighting the importance of further stratifying the regression analysis by sex. Our FFT model performance evaluation revealed a Misclassification Cost (MCU) of 2.1, with sensitivity at 48% and specificity at 57%, resulting in an overall accuracy of 54% and a balanced accuracy of 52%. These metrics suggest moderate performance in differentiating early from late-stage cancer.

# **Tables**

## **Table 1.** Characteristics of patients with known and unknown disease stage at CRC diagnosis

| Characteristic | | Known stage  N (%) | Missing stage  N (%) |
| --- | --- | --- | --- |
|  |  | 17541 (90.1) | 1922 (9.9) |
| Age in years | |  |  |
| 0-39 | | 1981 (11.3) | 208 (10.8) |
| 40-49 | | 3039 (17.3) | 270 (14.0) |
| 50-59 | | 4335 (24.7) | 417 (21.7) |
| 60-69 | | 4111 (23.4) | 457 (23.8) |
| 70-79 | | 2872 (16.4) | 314 (16.3) |
| 80+ | | 1196 ( 6.8) | 252 (13.1) |
| Mean age (SD) | | 57.9 (14.8) | 60.4 (16.5) |
| Sex | |  |  |
| Female | | 7898 (45.0) | 877 (45.6) |
| Male | | 9643 (54.9) | 1045 (54.4) |
| Marital status | |  |  |
|  | Married | 15847 (90.3) | 1781 (92.7) |
|  | Unmarried | 1694 (9.7) | 141 (7.3) |
| Region | |  |  |
| Riyadh | | 5370 (30.6) | 516 (26.8) |
| Eastern | | 2992 (17.1) | 308 (16.0) |
| Makkah | | 4350 (24.8) | 408 (21.2) |
| Madina | | 940 ( 5.4) | 185 ( 9.6) |
| Asir | | 1257 ( 7.2) | 137 ( 7.1) |
| Jazan | | 316 ( 1.8) | 37 ( 1.9) |
| Najran | | 168 ( 1.0) | 22 ( 1.1) |
| Hail | | 332 ( 1.9) | 69 ( 3.6) |
| Qassim | | 814 ( 4.6) | 100 ( 5.2) |
| Baha | | 255 ( 1.5) | 26 ( 1.4) |
| Jouf | | 145 ( 0.8) | 24 ( 1.2) |
| Northern | | 112 ( 0.6) | 23 ( 1.2) |
| Tabuk | | 355 ( 2.0) | 41 ( 2.1) |
| Anatomical site | |  |  |
| Colon | | 10540 (60.1) | 1089 (56.7) |
| Rectosigmoid | | 2720 (15.5) | 228 (11.9) |
| Rectal | | 4281 (24.4) | 605 (31.5) |

Note: N: Number; SD: Standard deviation.

## **Sensitivity analysis using multiple imputation for stage data in logistic regression**

## **Table *2*.** Adjusted odds ratios for late versus early-stage CRC presentation (N=19,463)

| Variable | OR (95%CI) |
| --- | --- |
| Age in years |  |
| 0-39 | 1.09 (0.98, 1.21) |
| 40- 49 | 1.12 (1.02, 1.22) |
| 50-59 | 1.00 *P=*0.018 |
| 60- 69 | 1.02 (0.93, 1.12) |
| 70-79 | 1.09 (0.98, 1.20) |
| 80+ | 1.08 (0.93, 1.22) |
| Sex |  |
| Male | 1.00 *P=*0.001 |
| Female | 1.12 (1.05, 1.18) |
| Marital status |  |
| Married | 1.00 *P=*0.08 |
| Unmarried | 1.06 (0.96, 1.17) |
| Region |  |
| Riyadh | 1.00 *P*<0.0001 |
| Eastern | 1.11 (1.01, 1.21) |
| Makkah | 0.91 (0.83, 1.00) |
| Madina | 0.82 (0.65, 1.00) |
| Asir | 0.91 (0.76, 1.06) |
| Jazan | 0.79 (0.53, 1.06) |
| Najran | 1.10 (0.76, 1.44) |
| Hail | 1.16 (0.93, 1.38) |
| Qassim | 1.06 (0.90, 1.22) |
| Baha | 0.70 (0.40, 1.00) |
| Jouf | 1.51 (1.18, 1.84) |
| Northern | 1.51 (1.14, 1.89) |
| Tabuk | 1.17 (0.95, 1.39) |
| Diagnosis date |  |
| 1997-2001 | 1.00 *P*<0.0001 |
| 2002-2006 | 1.23 (1.09, 1.37) |
| 2007-2011 | 1.34 (1.21, 1.48) |
| 2012-2017 | 1.27 (1.14, 1.40) |
| Anatomical site |  |
| Colon | 1.00 *P=*0.003 |
| Rectosigmoid | 1.16 (1.07, 1.25) |
| Rectal | 1.01 (0.94, 1.09) |

Note: CI: Confidence interval; N: Number; OR: odds ratios adjusted for all factors in the table using multiple imputation of covariates and disease stage. The p-values are derived from the overall likelihood ratio tests for association.

## **Table *3*.** Adjusted odds ratios for late versus early-stage CRC presentation, by sex (N=19,463)

| Variable | Males (N= 10,688) | | Females (N= 8,775) |
| --- | --- | --- | --- |
|  | **OR (95%CI)** | | **OR (95%CI)** |
| Age in years |  |  |  |
| 0-39 | 0.96 (0.79, 1.13) | | 1.22 (1.05, 1.38) |
| 40-49 | 1.04 (0.89, 1.18) | | 1.19 (1.05, 1.33) |
| 50-59 | 1.00 *P=*0.01 | | 1.00 *P=*0.006 |
| 60-69 | 0.95 (0.83, 1.08) | | 1.09 (0.94, 1.23) |
| 70-79 | 1.09 (0.95, 1.24) | | 1.06 (0.90, 1.23) |
| 80+ | 1.18 (0.99, 1.37) | | 0.92 (0.70, 1.14) |
| Marital status |  | |  |
| Married | 1.00 *P=*0.42 | | 1.00 *P=*0.01 |
| Unmarried | 1.12 (0.93, 1.31) | | 1.21 (1.07, 1.36) |
| Region |  | |  |
| Riyadh | 1.00 *P*<0.0001 | | 1.00 *P*<0.0001 |
| Eastern | 1.20 (1.06, 1.34) | | 1.03 (0.89, 1.18) |
| Makkah | 0.96 (0.84, 1.08) | | 0.87 (0.74, 1.00) |
| Madina | 0.90 (0.69, 1.11) | | 0.75 (0.52, 0.99) |
| Asir | 0.90 (0.71, 1.09) | | 0.97 (0.76, 1.17) |
| Jazan | 0.94 (0.59, 1.28) | | 0.63 (0.22, 1.04) |
| Najran | 1.33 (0.87, 1.79) | | 0.90 (0.41, 1.38) |
| Hail | 1.35 (1.04, 1.66) | | 0.89 (0.54, 1.24) |
| Qassim | 1.15 (0.93, 1.37) | | 0.94 (0.71, 1.18) |
| Baha | 0.80 (0.36, 1.23) | | 0.62 (0.21, 1.04) |
| Jouf | 1.86 (1.43, 2.28) | | 1.20 (0.69, 1.70) |
| Northern | 1.18 (0.63, 1.74) | | 1.87 (1.32, 2.41) |
| Tabuk | 1.25 (0.96, 1.55) | | 1.09 (0.75, 1.43) |
| Diagnosis date |  | |  |
| 1997-2001 | 1.00 *P=*0.001 | | 1.00 *P*<0.001 |
| 2002-2006 | 1.19 (0.99, 1.38) | | 1.26 (1.05, 1.48) |
| 2007-2011 | 1.34 (1.16, 1.52) | | 1.40 (1.20, 1.59) |
| 2012-2017 | 1.20 (1.03, 1.37) | | 1.39 (1.21, 1.57) |
| Anatomical site |  | |  |
| Colon | 1.00 *P=*0.02 | | 1.00 *P=*0.06 |
| Rectosigmoid | 1.17 (1.04, 1.30) | | 1.16 (1.02, 1.30) |
| Rectal | 0.99 (0.88, 1.09) | | 1.04 (0.92, 1.16) |

Note: CI: Confidence interval; N: Number; OR: odds ratios adjusted for all factors in the table using multiple imputation of covariates and disease stage. The p-values are derived from the overall likelihood ratio tests for association.

# **Figures**


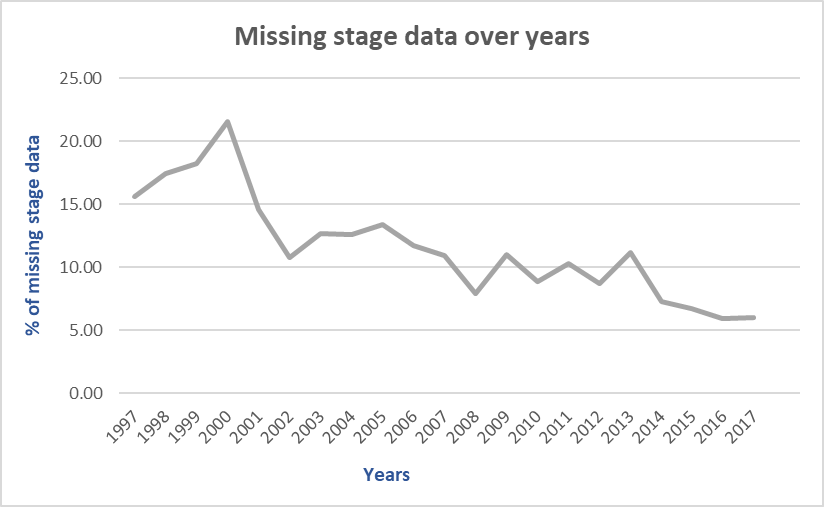


## **Figure 1.** Percentage of missing stage data for CRC in the Saudi Cancer Registry between 1997 and 2017


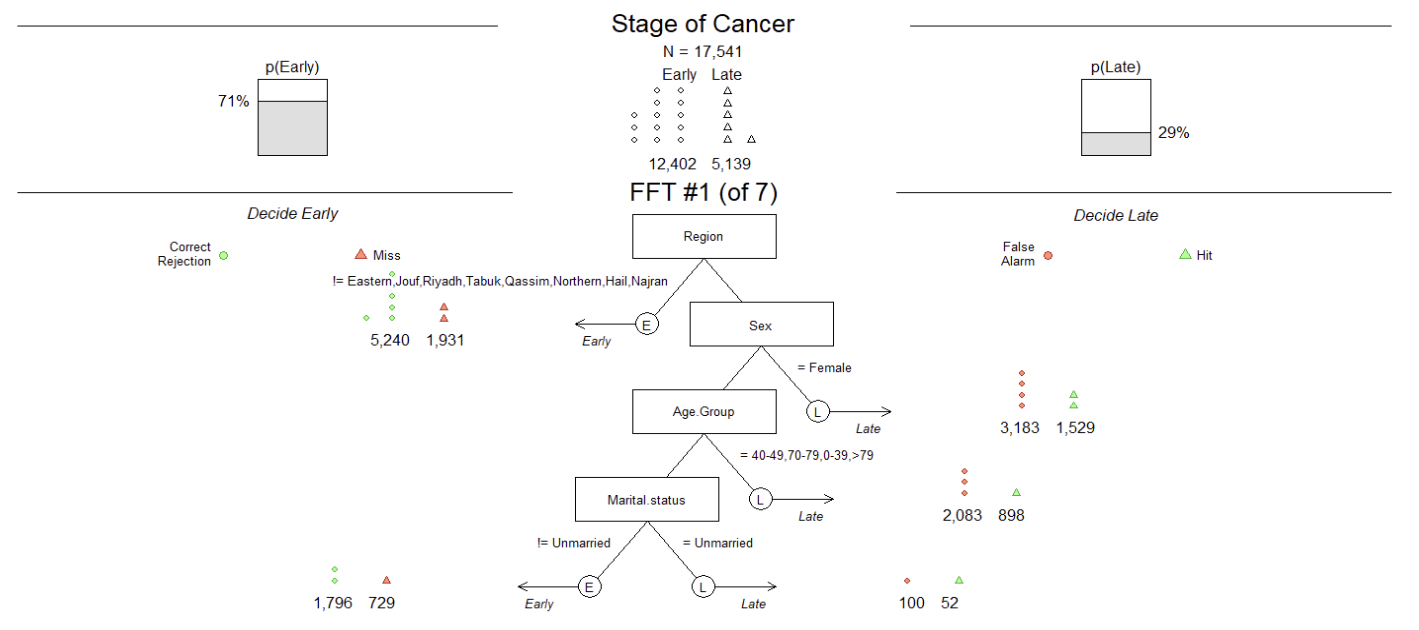


## **Figure 2.** A fast-and-frugal tree (FFT) for classifying patients as having late or early-stage CRC
